# Resting-State fMRI and Post-Ischemic Stroke Functional Recovery: Unraveling Causality and Predicting Therapeutic Targets

**DOI:** 10.3390/ijms26083608

**Published:** 2025-04-11

**Authors:** Mu-Zhi Li, Yin-Li Shi, Xiao-Jun He, Si-Cun Wang, Jun Liu, Zhong Wang, Hai-Xia Dang, Ya-Nan Yu

**Affiliations:** 1 Institute of Basic Research in Clinical Medicine, China Academy of Chinese Medical Sciences, Beijing 100700, China; 18473702850@163.com (M.-Z.L.); viviennne@foxmail.com (Y.-L.S.);; 2 China Academy of Chinese Medical Sciences, Beijing 100700, China

**Keywords:** Mendelian randomization analysis, post-ischemic stroke functional recovery (PISFR), rs-fMRI, ischemic stroke, drug target, protein pathway

## Abstract

Research on functional recovery after ischemic stroke has primarily focused on non-invasive brain stimulation and motor rehabilitation therapies, while direct pharmacological interventions are relatively underexplored. This study utilized a bidirectional Mendelian randomization approach to investigate the causal relationship between 191 resting-state functional magnetic resonance imaging (rs-fMRI) features and post-ischemic stroke functional recovery (PISFR). Significant rs-fMRI phenotypes were identified, and Mendelian randomization was employed to determine their associated proteins. Bidirectional Mendelian randomization identified four rs-fMRI phenotypes potentially associated with functional recovery after ischemic stroke. Subsequent MR analysis, using pheno12 as the outcome and plasma protein as the exposure, highlighted Fas-Associated protein with Death Domain (FADD) as a significant protein. Further exploration within the protein–protein interaction (PPI) network identified FADD, Cysteinyl Aspartate Specific Proteinase 8 (CASP8), and Receptor-Interacting Serine/Threonine-Protein Kinase 1 (RIPK1) as potential drug targets. Gene Ontology (GO) and Kyoto Encyclopedia of Genes and Genomes (KEGG) analyses indicated that these proteins are involved in the extrinsic apoptotic pathway, providing new insights for pharmacological strategies in post-ischemic stroke recovery. This study offers genetic evidence linking rs-fMRI to functional recovery post-ischemic stroke and identifies potential drug targets that may facilitate therapeutic interventions to enhance recovery after ischemic stroke.

## 1. Introduction

Ischemic stroke (IS) is a form of stroke characterized by acute, focal neurological deficits resulting from acute arterial occlusion [1]. In 2021, there were 93.8 million prevalent and 11.9 million incident strokes [2]. The global incidence of IS reached 7.63 million cases. Survivors of ischemic stroke often encounter challenges such as hemiparesis, aphasia, and impaired mobility, significantly impacting their ability to perform daily activities independently [3]. At present, approximately 100,000 individuals in the United Kingdom are living with post-stroke effects [4]. Even younger patients who have experienced mild ischemic strokes may face psychological issues, often struggling with depression and anxiety [5]. Consequently, post-ischemic stroke functional recovery (PISFR) has emerged as a critical area of research. To improve patients’ quality of life and promote PISFR, researchers have explored various strategies to support and preserve neurological function. Addressing long-term arm function loss following ischemic stroke, Dawson et al. (2021) implemented a therapeutic approach combining vagus nerve stimulation with rehabilitation, utilizing cholinergic and monoaminergic modulation of motor cortex neurons to enhance the brain’s reorganization potential [6]. In another study, Chollet et al. (2011) compared the efficacy of fluoxetine combined with physiotherapy to that of a placebo plus physiotherapy [7]. These studies primarily employed therapeutic approaches consisting of transcranial electrical stimulation and motor recovery interventions, often complemented by neurotrophic drug treatments. Compared to monotherapy, the time and economic costs associated with these combination treatment regimens significantly impact patient adherence [8]. However, targeted pharmacological treatments specifically promoting the recovery of functionally related neural pathways are currently unavailable [9].

Magnetic resonance imaging (MRI) is increasingly recognized as a crucial outcome measure in clinical studies, enabling researchers to assess the extent of neural recovery in the brain [10,11]. It is a primary diagnostic imaging modality that generates detailed structural images of the brain. However, the relationship between brain structure and function is nonlinear, and functional brain networks underpin the complex functions of the human brain. Consequently, analyzing brain function solely based on structural observations is insufficient. Resting-state functional magnetic resonance imaging (rs-fMRI) addresses this limitation. Rs-fMRI is a technique used to investigate spontaneous neural activity, characterized by its macroscopic organization and coherence [12]. This method utilizes ultrafast imaging techniques to capture functional information associated with changes in blood flow or oxygenation levels, thereby revealing the intricate networks of the brain at rest [13]. It has the capability to visualize interconnected regions of neural function within the brain and can demonstrate alterations in specific neural functional networks and their interactions with particular proteins [14,15].

Previous studies have demonstrated alterations in static and dynamic functional network connectivity in patients with post-stroke cognitive impairment compared to healthy individuals [16]. This finding indicates the presence of abnormal brain functional connectivity associated with functional impairment following a stroke. In the pathogenesis of ischemic stroke, various cell types in the central nervous system undergo distinct morphological changes in response to ischemic damage, disrupting the normal functioning of multiple bodily systems [17]. Due to neuroplasticity, the effects of ischemic stroke are partially reversible, a perspective supported by findings from various animal models [18,19,20]. As a promising biomarker for monitoring cognitive recovery after ischemic stroke [21], alterations in rs-fMRI may offer insights for identifying potential drug targets that directly influence functional connectivity networks related to PISFR. However, there is currently no causal evidence to determine whether changes in the brain’s functional networks result from functional recovery or whether they influence the recovery process. Understanding the causal relationship between these factors would enhance our comprehension of the neural mechanisms underlying post-ischemic stroke functional recovery, providing a foundation for research on potential therapeutic targets for functional recovery after stroke.

**Figure 1:**
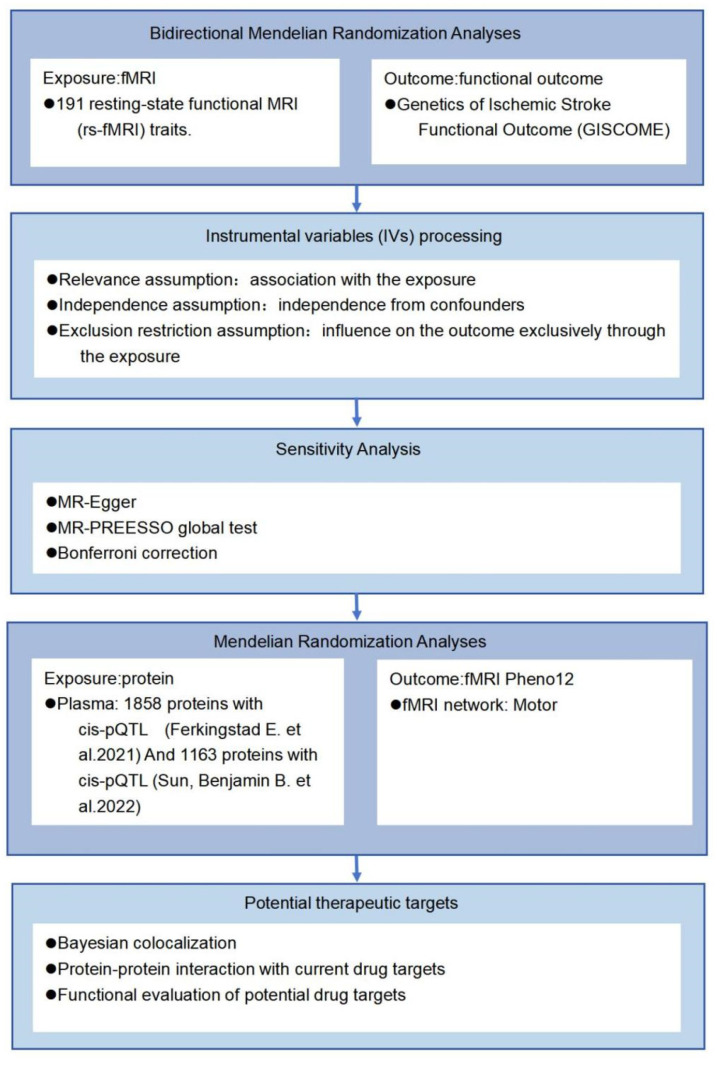
A flow chart of the study design. Single nucleotide polymorphisms (SNPs) significantly associated with resting-state functional magnetic resonance imaging (rs-fMRI) phenotypes and independent of confounding factors were utilized as instrumental variables (IVs). Plasma data were obtained from the studies conducted by Ferkingstad et al. [24] and Sun, Benjamin B. et al. [25].

Mendelian randomization (MR) is an instrumental variable analysis technique used to examine causal hypotheses in non-experimental data [22]. This approach can mitigate confounding bias and reverse causation bias due to the immutable nature of genetic instruments [23]. We conducted a two-sample Mendelian randomization analysis to investigate the causal relationship between PISFR and 191 rs-fMRI phenotypes, while simultaneously exploring potential drug targets within the relevant brain functional networks. Figure 1 presents an overview of the study design. This study presents the first systematic MR investigation of the causal relationship between rs-fMRI phenotypes and PISFR. The findings reveal potential biomarkers and pathways related to PISFR, offering novel insights into the mechanisms underlying functional recovery outcomes following IS.

The remainder of the paper is organized as follows: Section 2 introduces the materials and primary methods employed, including data sources, bidirectional two-sample MR analyses, and sensitivity analyses, among others. Section 3 provides a concise description and visualization of the main results. Section 4 analyzes and discusses the findings, while the concluding section articulates the key conclusions and outlines the potential for further analyses.

## 2. Results

### 2.1. Forward MR

From the initial GWAS summary statistics of 1770 rs-fMRI traits, 191 phenotypes significantly influenced by genetic variants (*p*_(IVs)_ < 5 × 10^−8^) were selected for further analysis. The comprehensive datasets of these fMRI phenotypes are available in the original publication and summarized in Supplementary Table S2. This investigation explored the causal relationship between the 191 rs-fMRI traits and functional outcomes following IS. The IVs employed in this study are detailed in Supplementary Table S3.

**Figure 2:**
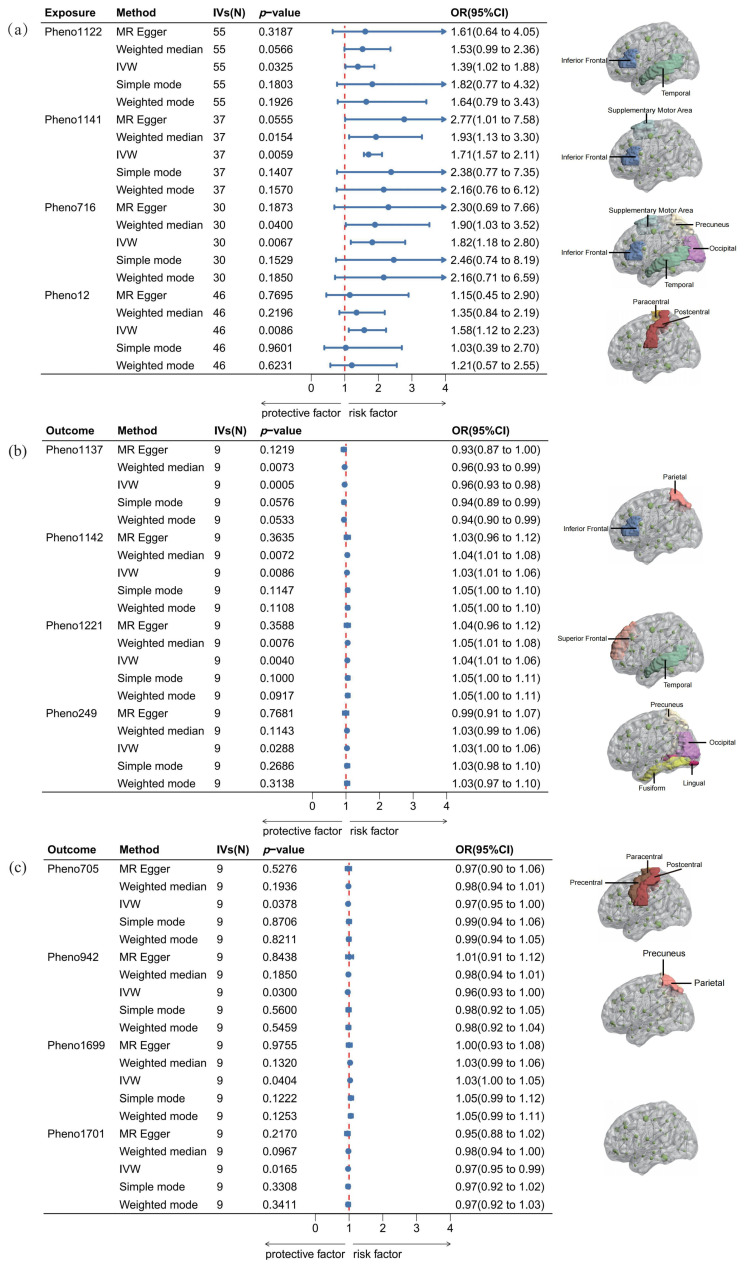
Causalities in the bidirectional MR. (**a**) The results of forward MR, (**b**) and (**c**) present the results of reverse MR. This study illustrates the causal relationships derived from bidirectional MR analysis using five methods: MR-Egger, weighted median, IVW, simple mode, and weighted mode. OR denotes odds ratio. In the forest plot, blue segments represent the confidence intervals for the OR, while blue dots indicate the OR values. The red line denotes the threshold for the OR. An OR value to the left of the red line (OR < 1) signifies a positive correlation, whereas an OR value to the right (OR > 1) indicates a negative correlation between the activity of the brain functional connectivity region and functional recovery after IS. A 95% CI represents the range of certainty for the estimated OR.

The bidirectional analyses employed an evidential threshold of *p*_(MR)_ < 0.05, identifying four rs-fMRI phenotypes (Pheno12, Pheno1122, Pheno1141, Pheno716) in the forward MR analysis. However, these associations lost significance after Bonferroni correction was applied to account for multiple comparisons (*p*_(adjMR)_ < 1.30 × 10^−4^). This indicates that there is insufficient causal evidence to establish a relationship between functional recovery after ischemic stroke and the 191 rs-fMRI phenotypes. Although the results did not reveal a statistically significant correlation after multiple testing adjustments, they suggest a potential avenue for further exploration and investigation. As shown in Figure 2, all four screened phenotypes were associated with poorer functional outcomes. Pheno12 is related to the activity of paracentral and postcentral regions, primarily affecting the motor function network. Our study indicated that an increase of one standard deviation (s.d.) in the amplitude trait of the paracentral and postcentral regions was associated with a 58% higher risk of functional recovery [IVW OR = 1.58, 95% confidence interval (CI): 1.12–2.23, *p*_(MR)_ = 0.0086]. Pheno1122 is related to the activity of temporal and inferior frontal regions and mainly affects the default mode network and the central executive network (or default mode network). Our findings show that an s.d. increase in the amplitude trait of the temporal and inferior frontal regions was associated with a 39% higher risk of functional recovery (IVW OR = 1.39, 95% CI: 1.02–1.88, *p*_(MR)_ = 0.0325). Pheno1141 represents the activity of the supplementary motor area and inferior frontal region and is connected with the salience network (or default mode network) and the central executive network (or default mode network). An s.d. increase in this functional connectivity increased the risk of functional recovery after ischemic stroke by 71% (IVW OR = 1.71, 95% CI: 1.57–2.11, *p*_(MR)_ = 0.0059). Pheno716 is connected with the occipital lobe (or precuneus) and temporal (or frontal, supplementary motor area) regions. The MR analysis showed that the activity of these brain regions affected the functional connectivity in the default mode (or central executive) network and default mode (or salience) network. A one s.d. increase in the functional connectivity was associated with an 82% higher risk of functional recovery (IVW OR = 1.82, 95% CI:1.18–2.80, *p*_(MR)_ = 0.0067). The detailed results of the forward MR can be found in Table 1 and Figure 2, Figure S2.

### 2.2. Reverse MR

Previous studies suggest that functional recovery after IS may be associated with nerve repair [26]. To further investigate this relationship, we conducted a reverse MR analysis to examine the potential effects of functional restoration on brain functional networks. Eight rs-fMRI phenotypes (Pheno1137, Pheno1142, Pheno1221, Pheno705, Pheno249, Pheno942, Pheno1699, Pheno1701) were identified in the reverse MR analysis. The IVs employed in this study are presented in Supplementary Table S4. The research suggests that functional recovery following ischemic stroke may influence various brain networks, including the visual, motor, triple, default mode, central executive, and salience networks. However, caution is warranted in interpreting these findings, as the observed statistical significance does not survive Bonferroni correction. Detailed results are presented in Table 2 and Figure 2. The OR values for these associations are all close to one, indicating that the impact of post-ischemic stroke functional recovery on these brain functional connectivity regions is minimal. Additionally, this analysis reveals no causal effect on the brain functional connectivity regions identified in our forward MR analysis.

### 2.3. Sensitivity Analyses for rs-fMRI Associated with Functional Outcome After Ischemic Stroke

A series of sensitivity analyses were conducted to demonstrate the robustness of the forward MR analysis. Initially, we measured the MR-Egger intercepts of the four identified phenotypes, and none exhibited significant horizontal pleiotropy (*p*_(egger)_ > 0.05). Subsequently, the MR-PRESSO global test was performed, further supporting the aforementioned findings. Although our study is a two-sample MR analysis, both datasets are based on European populations; therefore, we conducted a Cochran’s Q test, which revealed no significant heterogeneity in the study. Additionally, a leave-one-out analysis was performed, sequentially excluding each SNP and calculating the meta-effects of the remaining SNPs to assess whether the results changed upon the removal of any specific SNP. The visualization of these results is presented in Figure S1. The findings indicate that after removing any single SNP, the direction of beta remains consistent, and the causal relationship between exposure and outcome persists. Detailed results of the sensitivity analysis can be found in Table 1 and Table 2.

### 2.4. Identification of rs-fMRI-Associated Proteins

**Figure 3:**
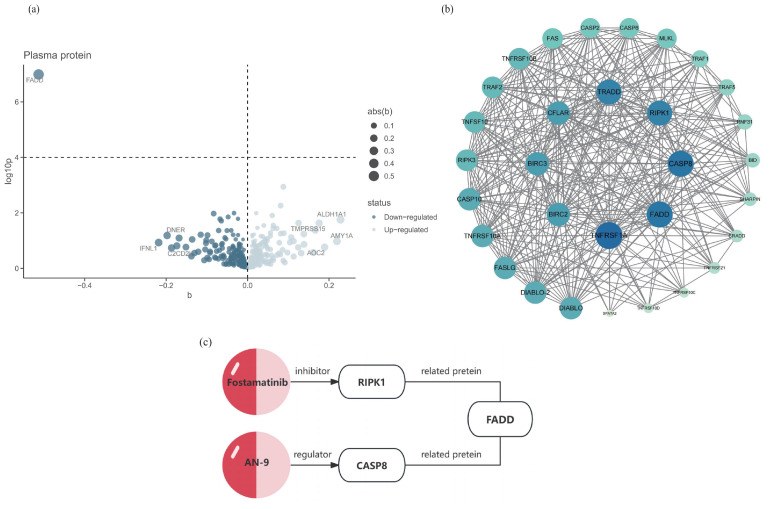
Potential drug targets of Pheno12 and its interacting proteins. (**a**) MR results for plasma protein and functional recovery after IS. This volcano plot illustrates the MR results between 494 plasma proteins and Pheno12; the MR analysis methodology employs either the IVW approach or the Wald ratio; the black horizontal dashed line corresponds to a *p* = 0.0001 (0.05/494). (**b**) The PPI network of the pathogenic protein FADD, with darker colors indicating a higher number of associated nodes. Darker colors indicate a stronger correlation between the proteins. (**c**) The FADD-associated proteins RIPK1 and CASP8, along with their respective targeted drugs.

Among the four significant phenotypes identified, Pheno12 is associated with the motor function network. The recovery of motor function after stroke has consistently been a primary concern in post-stroke functional recovery, as patients with impaired functionality often experience significant distress when performing daily activities such as bathing and grooming [27]. Consequently, an additional MR analysis was conducted to assess the evidence for a causal relationship between the 494 significant proteins and Pheno12. Fas-Associated protein with Death Domain (FADD) was the only protein identified to have a causal relationship with this phenotype; the visualization results are presented in Figure 3a. The analysis demonstrated that FADD has an inhibiting effect on the motor function network (IVW OR = 1.51, 95% CI: 0.81–1.19, *p*_(MR)_ = 1.03 × 10^−7^). The sensitivity analysis is provided in Supplementary Table S5, Figure S3.

### 2.5. Colocalization Analysis and Functional Evaluation of Potential Drug Targets

**Figure 4:**
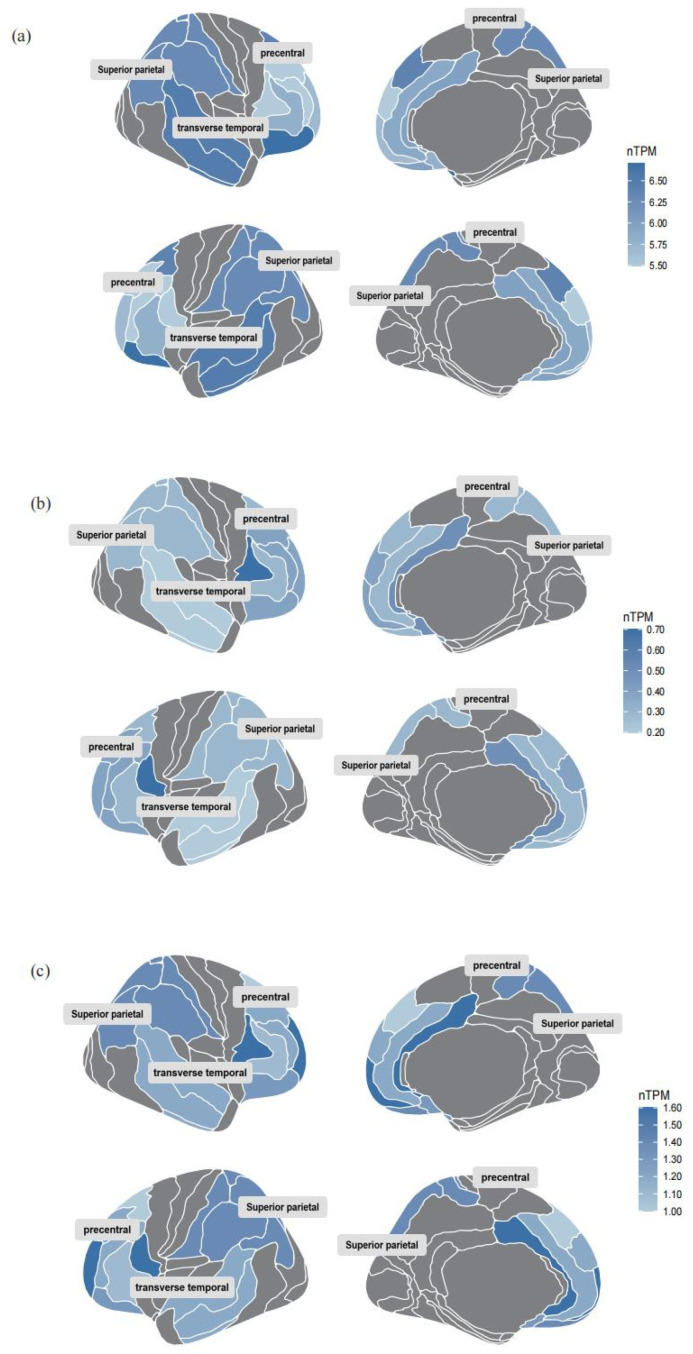
Brain RNA expression of the three identified proteins. (**a**) Brain RNA expression of FADD. (**b**) Brain RNA expression of CASP8. (**c**) Brain RNA expression of RIPK1. The figure presents data from a subset of brain regions, with darker colors indicating higher protein expression levels.

Colocalization analysis of the screened proteins FADD and Pheno12 was performed using the coloc package, demonstrating strong evidence of colocalization (PP.H4 = 0.9991, Supplementary Table S6). The eQTLs data for FADD were obtained from the cis-eQTLs data in the eQTL database. The target gene of FADD was input into the STRING database, generating a PPI network consisting of 31 nodes and 337 edges. These results were then imported into Cytoscape for visual analysis (Figure 3b). A search of the DrugBank database did not identify any drugs targeting FADD directly; therefore, based on the PPI network, the search scope was expanded to include predicted functional partners of FADD. This approach identified two relatively well-established drug targets: CASP8 and RIPK1. These proteins are hypothesized to potentially influence the motor function network through protein interactions, thereby affecting motor function recovery following ischemic stroke. According to the Human Protein Atlas database [28], the expression of these three identified proteins in the brain is illustrated in Figure 4.

**Figure 5:**
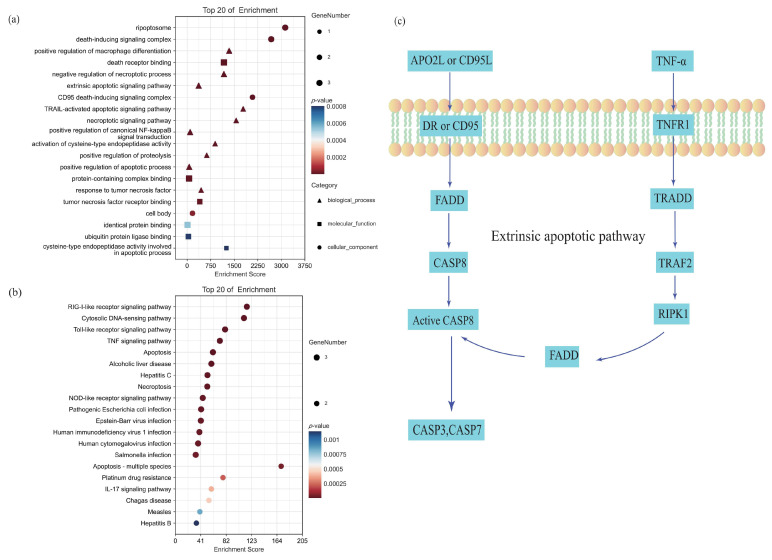
Common pathway of the three potential targets. (**a**) GO analysis of the three identified genes. (**b**) KEGG pathway enrichment analysis of the three identified genes. (**c**) Common pathways of the three identified proteins from the KEGG database.

A comprehensive analysis of the GO and KEGG databases revealed that the three identified genes play a crucial role in the formation of the death-inducing signaling complex, which is closely associated with apoptosis and necrosis processes (Figure 5a,b). The KEGG database results indicate that these three proteins are located within the extrinsic apoptotic pathway (Figure 5c), which is fundamental to apoptosis and cancer pathogenesis. Specifically, death receptors facilitate the recruitment and activation of the initiator caspase CASP8 via FADD, thereby initiating the apoptotic process. Additionally, RIPK1 is involved in the canonical NF-κB signaling pathway, where it regulates the expression of apoptosis-associated factors. Non-ubiquitinated RIP1 readily interacts with FADD and CASP8 to form a secondary complex that propagates a receptor-independent death signal, thus promoting cell death progression.

## 3. Discussion

Bidirectional two-sample Mendelian randomization analyses identified four rs-fMRI phenotypes (Pheno12, Pheno1122, Pheno1141, Pheno716) with potential causal effects on PISFR. Elevated signals in these phenotypes were associated with increased risks of PISFR, providing causal evidence for the relationship between brain rs-fMRI and PISFR. The identification of these specific brain functional connectivity areas enables the implementation of more targeted repetitive transcranial magnetic stimulation (rTMS) or non-invasive brain stimulation (NIBS) interventions in future research. Furthermore, drug target prediction for Pheno12, which is of particular interest, revealed a potential druggable protein along with two related proteins. This discovery may offer new avenues for pharmacological treatments targeting this specific area of brain functional connectivity.

Functional imaging has been employed as a biomarker in stroke-related clinical research for decades [29]. Previous studies have shown that functional connectivity between temporal and inferior frontal regions is likely associated with cognitive functions related to language comprehension and text processing [30]. The co-activation of the inferior frontal gyrus (IFG) and the posterior middle temporal gyrus (pMTG) is typically linked to controlled semantic retrieval of words [31]. After a stroke, some patients may experience aphasia, characterized by impaired language and cognitive abilities, primarily associated with functional abnormalities in the left supramarginal gyrus and the left or bilateral precunei of the brain [32]. This MR analysis indicates that temporal and inferior frontal regions are negatively correlated with functional outcomes following stroke. In light of previous studies, these regions may be associated with aphasia following ischemic stroke.

Following subcortical motor stroke, patients frequently exhibit increased activations in secondary motor regions, including the dorsal premotor cortex (PMd), ventral premotor cortex, supplementary motor area (SMA), and cingulate motor area in both the affected and unaffected hemispheres, as well as the contralesional primary motor cortex [33]. Evidence suggests that enhanced regional connectivity of the SMA and IFG may impede motor function recovery after IS [34,35]. The forward MR results support this perspective, indicating that increased activity in the SMA and the IFG negatively impacts functional recovery post-stroke.

Numerous studies have demonstrated that the occipitotemporal cortex plays a crucial role in human visual functions [36,37]. This region receives visual feature information transmitted from the visual cortex and computes the canonical size of objects. Following a stroke, patients may experience visual field loss, a functional impairment that can significantly impact their mobility and ability to judge distances [38]. Previous research has identified that patients with visual field defects after acute stroke exhibit notable increases in early resting-state functional connectivity (RSFC) within the visual cortex, which contrasts with our MR results [39]. However, the interpretation of these findings may be influenced by the imbalance of interhemispheric blood supply post-stroke. Consequently, further research is necessary to elucidate the causal relationship between these factors.

To further investigate the potential for pharmacological treatment of motor function recovery following IS, an MR analysis with protein exposure was conducted. Through our examination of drug target prediction for the neural connectivity function of the paracentral and postcentral regions, following colocalization analysis, we found strong evidence that a higher level of FADD predicted by genetics is negatively associated with this connectivity area. FADD has consistently been a prominent research subject for targeted drug therapies, serving as a promising clinical prognostic biomarker and therapeutic target for cancer patients [40]. To explore this further, we identified ten predicted functional partners of FADD (TNFRSF1A, RIPK3, TNFRSF10A, TRAF2, RIPK1, TNFRSF10B, CASP10, CFLAR, TRADD, CASP8) through the association of the PPI network, which have strong interaction relationships with FADD. Through a comprehensive search of the DrugBank database, two proteins were ultimately selected based on the availability of corresponding target drugs that have successfully passed clinical trial safety validations (Figure 3c). A review of the literature reveals that FADD is an adaptor molecule that interacts with various cell surface receptors and mediates cell apoptotic signals [41]. It primarily acts on the prefrontal cortex, striatum, and hippocampus of the brain [42]. CASP8 is a protease with both pro-death and pro-survival functions, expressed at very low levels in the brain, making it nearly undetectable [43]. RIPK1 is a protein kinase that regulates homeostasis at the cellular and tissue levels by integrating inflammation and cell death signaling pathways, and it is widely expressed in various brain regions [44].

CASP8 serves as a crucial molecular switch for apoptosis, necroptosis, and pyroptosis in programmed cell death [45]. In zebrafish embryos, the upregulation of apoptosis-related genes associated with CASP8 results in shortened motor neuron axons and impaired electrophysiological neural function [46]. Moreover, research suggests that the activation of CASP proteins can mediate hypoxic–ischemic brain damage in newborns, leading to deficits in motor and sensory functions [47]. These findings collectively demonstrate CASP8’s potential impact on brain functional connections affecting motor function. Pivanex (AN-9), a histone deacetylase inhibitor analog of butyric acid, functions as a regulator of the CASP8 protein [48]. It has shown promise in the treatment of non-small cell lung cancer (NSCLC), exhibiting low toxicity and significant anticancer activity both in vitro and in vivo [49]. While pivanex is considered relatively non-toxic and safe as a selective drug for malignant tumors, its application to neural recovery of brain function necessitates further ethical consideration.

The kinase structure of RIPK1 is highly conducive to the development of specific pharmacological small molecule inhibitors, making it a prevalent target in pharmaceutical research [50]. RIPK1 kinase-dependent necroptosis can elicit a potent inflammatory response, significantly alter the surrounding tissue environment, and influence the pathogenesis of central nervous system disorders. Consequently, it has emerged as a key therapeutic target for candidates currently undergoing clinical trials for neurological diseases [51]. In light of our findings, we posit that RIPK1 has substantial potential as a drug target for brain functional connectivity regions. Fostamatinib, a widely used RIPK1 inhibitor in clinical practice, has shown promising results in a randomized trial for the treatment of immune thrombocytopenia (ITP), demonstrating clinically significant responses in patients [52]. Moreover, a meta-analysis indicates that Fostamatinib is safe for the cardiovascular system [53]. Considering these factors, and given that Fostamatinib’s safety profile has been validated through multiple clinical trials and real-world applications, it may offer greater potential as a treatment target for the neural functional network in this brain region compared to AN-9.

Through the analysis of the GO and KEGG databases for the three identified proteins, this study elucidated the pathways through which they interact collectively. The research findings suggest that inhibition of the extrinsic apoptotic pathway by suppressing protein expression may promote post-stroke functional recovery. Additionally, the identification of the common pathway provides novel insights for the development of therapeutic agents aimed at enhancing functional recovery.

This study employed an MR analysis method, utilizing genetic factors as instrumental variables, which significantly mitigated the influence of confounding factors and provided more robust causal inferences. Additionally, rs-fMRI data were utilized for analysis. Compared to structural MRI data, rs-fMRI provides a more detailed picture of neural activity involved in complex brain functions, directly reflecting the functional activity intensity of different brain functional connectivity regions. The GWAS data used in the study were all derived from European populations, which to some extent reduced the impact of population stratification.

Nevertheless, this study has several limitations. Primarily, the MR analysis conducted in this study is limited to inferring the linear causal relationship between brain functional networks and functional recovery following ischemic stroke. Machine learning methods, an essential aspect of artificial intelligence, include emerging causal inference tools such as random forests [54] and elastic nets [55]. The application of these tools in MR analysis can effectively address the sparsity of high-dimensional data and facilitate the exploration of interactions and nonlinear relationships between genes and phenotypes, thereby enabling the identification of additional druggable gene targets. Moreover, the GWAS data were derived solely from individuals of European ancestry, restricting the generalizability of the findings. The protein target prediction analysis utilized only cis-eQTL, omitting trans-eQTL, which may have affected the comprehensiveness of the results. Lastly, this exploratory study identified statistical causal relationships, with the evidence obtained being predominantly suggestive rather than conclusive. The applicability of these causal relationships to functional recovery following ischemic stroke necessitates further validation through rigorous clinical trials.

## 4. Materials and Methods

### 4.1. Data Sources

The datasets utilized in this study are publicly accessible. The rs-fMRI GWAS summary datasets originated from a study aimed at identifying and validating common genetic variants influencing intrinsic brain activity, encompassing 191 rs-fMRI traits (75 ICA resting node amplitude and 116 ICA resting edge) [56]. The study investigated the associations between 1777 intrinsic brain activity phenotypes from 47,276 subjects and 9,026,427 common variants from 34,691 subjects in the UK Biobank (UKB) [57]. The outcome data were obtained from the Genetics of Ischemic Stroke Functional Outcome (GISCOME) network [58]. This network included 6165 patients aged 18 years or above with ischemic stroke from 12 studies across Europe, the United States, and Australia. The modified Rankin Scale (mRS) score after 60 to 190 days served as the primary outcome. The summary genome-wide association studies (GWASs) of functional outcomes, assessed using the mRS three months post-ischemic stroke, were employed as the outcome data. In these datasets, mRS 0–2 indicated good functional outcome (n = 3741), while mRS 3–6 represented poor functional outcome post-stroke (n = 2280).

In the MR analysis of target prediction, protein quantitative trait loci (pQTL) data for plasma proteins were obtained from two distinct datasets. The first dataset comprises GWASs of plasma protein levels measured using 4907 aptamers in 35,559 Icelandic individuals from Decode [24]. The second dataset originates from the UK Biobank Pharma Proteomics Project, encompassing pQTL mapping of 1463 proteins from 54,306 UK Biobank participants [25]. A comprehensive overview of the datasets utilized in this study can be found in the original publication, with a summary provided in Supplementary Table S1.

### 4.2. Bidirectional Two-Sample MR Analyses

The analysis utilized GWAS data of 191 rs-fMRI phenotypes significantly associated with 9,026,427 common variants among 1777 intrinsic brain activity phenotypes in the UKB as exposures. Among the 191 phenotypes, 75 reflect the amplitude characteristics of local spontaneous neuronal activity, 5 represent global functional connectivity features, and the remaining 111 quantify interactions between brain functional connectivity regions through pairwise functional connectivity characteristics. The IVs utilized in MR must satisfy three core assumptions (Figure 1). Given the limited number of SNPs with significant genome-wide effects, genetic variants associated with rs-fMRI were selected as candidate IVs in the forward MR analysis, employing a genome-wide significance threshold of *p*_(IVs)_ < 5 × 10^−8^. In instances where the number of screened SNPs was relatively small under the traditional threshold, a relaxed statistical threshold (*p*_(IVs)_ < 1 × 10^−5^) is commonly applied in MR analysis [59]. Linkage disequilibrium (LD) estimation was conducted using the 1000 Genomes Project European data (Phase 3) to identify independent SNPs (r^2^ < 0.001, a window size of 1000 kb to 10,000 kb). For the second assumption, SNPs associated with PISFR were excluded. Based on previous research, sleep condition [60], depression, and anxiety [61,62] are associated with functional outcomes after IS, so SNPs highly correlated with these three phenotypes were removed using the FastTrait R package (https://www.ebi.ac.uk/gwas/ (accessed on 10 November 2024)). Furthermore, SNPs significantly associated with the outcome (*p*_(IVs)_ < 5 × 10^−8^) were designated for exclusion. The inverse-variance weighted (IVW) method was employed as the primary analysis to determine Mendelian randomization estimates. To augment and enhance the reliability of the results, four additional Mendelian randomization methods were implemented: MR-Egger, weighted median, simple mode, and weighted mode.

Given that functional injury can significantly impact brain function following IS [63], the MR analysis was expanded to investigate bidirectional causal relationships between rs-fMRI and post-IS functional outcomes. The forward MR analysis designated rs-fMRI as the exposure and post-IS functional outcome as the outcome. Conversely, the reverse MR analysis designated post-IS functional outcome as the exposure and rs-fMRI as the outcome. The odds ratio (OR) was employed to quantify the causal effect size between exposure and outcome. These MR analyses were performed using the TwoSampleMR (version 0.6.2) and ggplot2 (version 3.5.1) R packages.

### 4.3. Sensitivity Analysis

A total of 191 phenotypes were utilized as exposures, necessitating the conduct of multiple independent tests. To mitigate the risk of false positives arising from multiple comparisons, the significance threshold (*p*_(MR)_ < 0.05) was adjusted using Bonferroni correction [64]. Associations were deemed statistically significant following this correction. To assess heterogeneity in each MR analysis method, Cochran’s Q statistics were calculated [65]. Effect size estimates and their variances for each IV were used to compare the degree of deviation of individual IV effect sizes from the overall effect. A positive Cochran’s Q (*p*_(Q)_ < 0.05) indicates heterogeneity in the results of the MR analysis. To detect directional pleiotropy, MR-Egger regression was employed [66]. In this analysis, regression was performed using the exposure effect estimates, outcome effect estimates, and their standard errors for each IV, with *p*_(egger)_ < 0.05 indicating the presence of pleiotropy. The MR-PRESSO global test utilized the same variable values as MR-Egger regression to assess the presence of horizontal pleiotropy (*p*_(PRESSO)_ < 0.05) and to identify and remove outliers using the MR-PRESSO (version 1.0) package [67].

Lastly, leave-one-out analyses were conducted to determine whether a single SNP substantially influenced the causal association [68].

### 4.4. MR Analysis of Target Prediction

Several studies demonstrate that fMRI can serve as a biomarker for functional recovery following stroke [69,70,71]. These findings suggest that identifying drug targets within the corresponding brain functional network may reveal targets influencing PISFR by modulating this network. A drug target prediction MR analysis was conducted on the phenotypes of interest based on bidirectional two-sample Mendelian randomization analyses. The analysis utilized protein quantitative trait loci (pQTLs) from 4907 proteins in the Decode database and 1463 proteins from the UK Biobank as exposures, with a significant rs-fMRI trait as the outcome for Mendelian randomization analysis. The selection of IVs adhered to the same methodology as the bidirectional MR analyses, utilizing a genome-wide significance threshold of *p*_(IVs)_ < 5 × 10^−8^ to identify SNPs closely associated with whole blood proteins. Four MR analysis methods were employed: IVW, MR-Egger, weighted median, and simple mode, along with weighted mode. In cases where only a single genetic instrument was available, the Wald ratio was employed, followed by a heterogeneity analysis.

### 4.5. Colocalization Analysis

To evaluate whether linkage disequilibrium influences the causal relationship between the identified proteins and functional recovery after ischemic stroke, Bayesian colocalization analysis was employed to investigate shared causal variation between the two traits, thereby enhancing the robustness of the Mendelian randomization analysis results. This analysis, based on a Bayesian model, evaluated five exclusive hypotheses: (1) no association with either trait; (2) association with trait 1 only; (3) association with trait 2 only; (4) both traits are associated, but distinct causal variants underlie the two traits; and (5) both traits are associated and share the same causal variant.

In this study, we examined the posterior probability of hypothesis 5 (PP.H4) to determine if the protein and rs-fMRI share the same causal variation. Utilizing the “coloc” R package with default arguments (https://github.com/chr1swallace/coloc (accessed on 10 November 2024)), we defined strong evidence of colocalization as PP.H4 > 0.8, while 0.4 < PP.H4 < 0.8 was considered moderate colocalization [68].

### 4.6. Function Analysis of Potential Drug Targets

To investigate the associated proteins and their potential interactions with the identified proteins, the STRING database (https://string-db.org/ (accessed on 25 December 2024)) was employed to construct a protein–protein interaction (PPI) network. A minimum interaction score of 0.4 was used, with default settings applied for all other parameters. This approach aims to enhance the understanding of intracellular protein interactions [72]. To further assess the druggability of the identified proteins, a search was conducted in the DrugBank database (https://go.drugbank.com/ (accessed on 25 December 2024)) and the Human Protein Atlas database (Human Protein Atlas proteinatlas.org). It is important to note that the search for druggable proteins included not only the significant proteins from the MR analysis but also the related proteins with strong interactions in the PPI network. Cytoscape (version 3.10.2) was used to visualize the findings of this analysis.

Additionally, a functional analysis of the identified proteins was performed using the Gene Ontology (GO) and Kyoto Encyclopedia of Genes and Genomes (KEGG) databases to further investigate their potential mechanisms of action. GO is an analytical framework utilized to investigate the commonalities among genes in terms of biological processes (BP), molecular functions (MF), and cellular components (CC). It assesses gene enrichment for each GO annotation by comparing the analyzed genes to a reference genome, thereby producing results for enrichment analysis. The primary focus of KEGG enrichment analysis is the identification of gene enrichment within metabolic pathways. Enrichment analysis was performed on a set of proteins exhibiting positive colocalization results, filtering the outcomes to include only those proteins with a corrected *p*-value of less than 0.05.

### 4.7. Statistical Analysis

In this study, statistical significance for causal associations was defined as *p*_(MR)_ < 0.05. For the sensitivity analysis, the Bonferroni correction threshold was adjusted to *p*_(adjMR)_ < 1.31 × 10^−4^ (0.05/398), reflecting the number of fMRI samples. *p*-values between 1.31 × 10^−4^ and 0.05 were interpreted as suggestive evidence for potential causal associations [73]. All analyses were conducted using R software (version 4.4.0).

## 5. Conclusions

In summary, this study identified four potential causal relationships between 191 rs-fMRI phenotypes and functional recovery after IS. These four rs-fMRI phenotypes (Pheno12, Pheno1122, Pheno1141, Pheno716) indicate the presence of brain functional networks that may influence functional recovery and have the potential to serve as biomarkers for recovery following ischemic stroke. Building on these results, the study also identified the related protein FADD and its associated proteins CASP8 and RIPK1 as potential drug targets for enhancing functional connectivity in the paracentral and postcentral brain regions. These findings may enhance the understanding of the cerebral mechanisms underlying functional recovery after ischemic stroke, offering additional avenues for pharmacological treatment in patients with visual, language, and motor impairments following the event.

## Figures and Tables

**Table 1:** Sensitivity analyses for significant rs-fMRI in forward MR.

Exposures	Pleiotropy (IVW)			Heterogeneity (IVW)		MR-PRESSO
	Egger_Intercept	se	*p*-Value	Cochran’s Q	*p*-Value	*p*-Value
Pheno12	0.198	0.027	0.471	49.671	0.258	0.276
Pheno716	−0.013	0.031	0.689	20.903	0.829	0.840
Pheno1122	−0.008	0.024	0.746	47.440	0.577	0.588
Pheno1141	−0.026	0.025	0.318	36.045	0.419	0.451

**Table 2:** Sensitivity analyses for significant rs-fMRI in reverse MR.

Outcomes	Pleiotropy (IVW)			Heterogeneity (IVW)		MR-PRESSO
	Egger_Intercept	se	*p*-Value	Cochran’s Q	*p*-Value	*p*-Value
Pheno249	0.013	0.012	0.314	8.865	0.354	0.401
Pheno705	0.0003	0.012	0.977	7.909	0.442	0.478
Pheno942	−0.014	0.015	0.370	14.489	0.070	0.095
Pheno1137	0.008	0.011	0.522	4.797	0.780	0.814
Pheno1142	−0.001	0.011	0.905	3.285	0.915	0.921
Pheno1221	−0.0005	0.011	0.963	5.489	0.704	0.742
Pheno1699	0.007	0.011	0.540	9.440	0.307	0.340
Pheno1701	0.007	0.011	0.561	6.401	0.602	0.599

## Data Availability

GWAS summary statistics for GISCOME are available at https://www.strokegenetics.org/ (accessed on 20 June 2024). The eQTL summary data from Decode were accessed via https://www.decode.com/summarydata/ (accessed on 20 June 2024). GWAS summary statistics for rs-fMRI were obtained from https://doi.org/10.5281/zenodo.5775047. Protein expression data were sourced from the Human Protein Atlas, v23.0.proteinatlas.org, accessed on 1 September 2024. All of the aforementioned data are freely accessible on the website.

## Supplementary Materials

The following supporting information can be downloaded at: https://www.mdpi.com/article/10.3390/ijms26083608/s1.
