# Supplementary material for: Resting-State fMRI and Post-Ischemic Stroke Functional Recovery: Unraveling Causality and Predicting Therapeutic Targets
Source: Int J Mol Sci. 2025 Apr 11;26(8):3608. doi: 10.3390/ijms26083608 (PMC12027196; doi:10.3390/ijms26083608)

Figure S1 Leave-one-out analyses of identified phenotypes. The y-axis corresponds to each excluded SNP, with the line segments representing the confidence interval of the beta values. (A)Pheno12, (B)Pheno716, (C)Pheno1122, (D)Pheno1141.

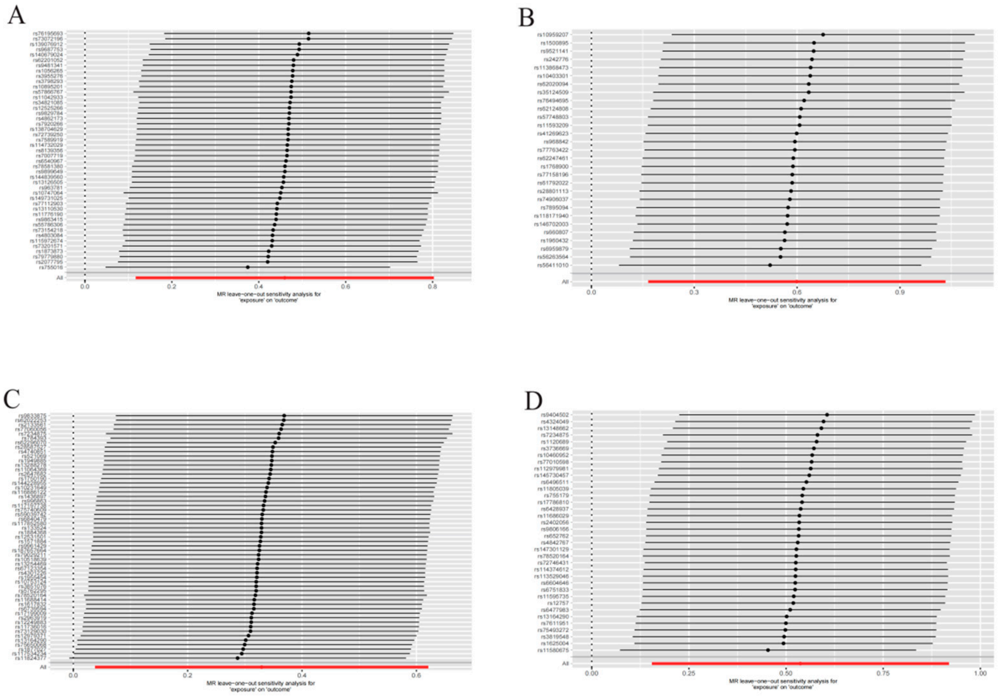

Figure S2 Scatter plot of identified phenotypes. (A)Pheno12, (B)Pheno716, (C)Pheno1122, (D)Pheno1141.

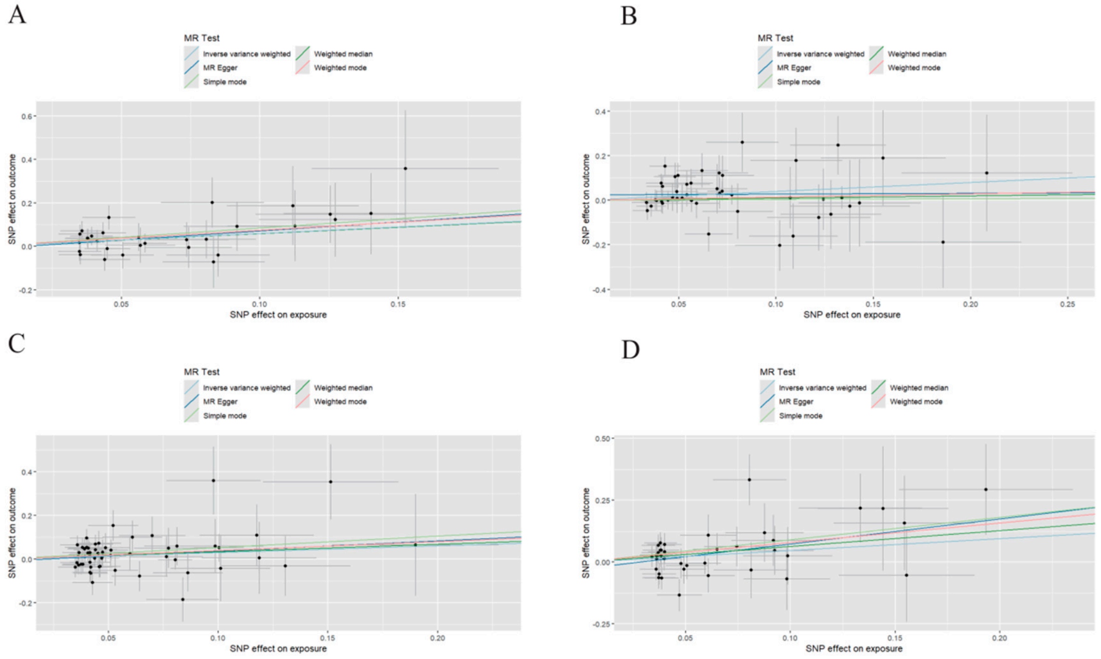

Figure S3 Leave-one-out analyses of identified proteins. The y-axis corresponds to each excluded SNP, with the line segments representing the confidence interval of the beta values.

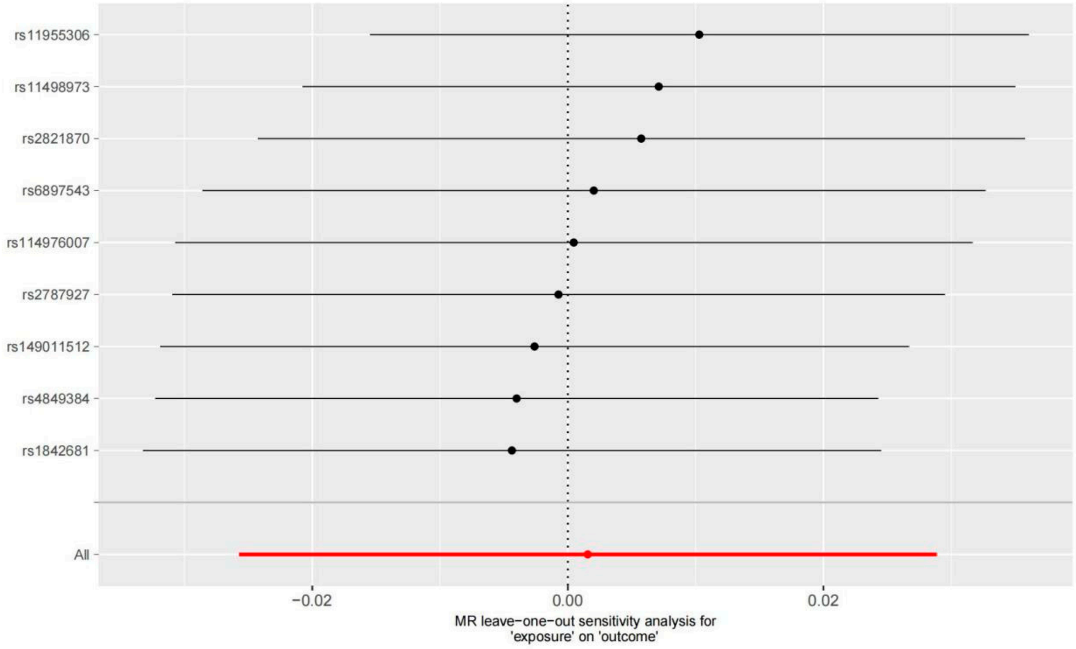

Supplement: Supplementary file 1 [file ijms-26-03608-s001.zip › supplement picture.pdf]
